# Factors related to nurses’ posttraumatic growth during the early stage of the coronavirus disease 2019 pandemic: a scoping review

**DOI:** 10.1093/joccuh/uiaf030

**Published:** 2025-05-26

**Authors:** Kana Sato, Keiko Ishii, Satoko Nagai, Yasuko Ogata

**Affiliations:** Faculty of Nursing, Mie Prefectural College of Nursing, 1-1-1, Yumegaoka, Tsu-shi, Mie 514-0116, Japan; Home Health and Palliative Care Nursing, Graduate School of Health Care Sciences, Institute of Science Tokyo, Bunkyo-ku, Tokyo, Japan; Department of Intensive Care Medicine, Graduate School of Medical and Dental Sciences, Institute of Science Tokyo, Bunkyo-ku, Tokyo, Japan; Nursing Innovation Science, Graduate School of Health Care Sciences, Institute of Science Tokyo, 1-5-45, Yushima, Bunkyo-ku, Tokyo 113-8519, Japan

**Keywords:** posttraumatic growth, COVID-19, nursing, scoping review, trauma

## Abstract

**Objectives:**

This study aimed to identify the factors influencing nurses’ posttraumatic growth (PTG) during the early stages of the coronavirus disease 2019 (COVID-19) pandemic.

**Methods:**

A literature search was conducted in February 2023 across databases, including Medline, CINAHL, APA PsycINFO, Web of Science, and Google Scholar, for articles published between January 2020 and February 2023 related to PTG in nurses during the COVID-19 pandemic. Inclusion criteria were English-language articles, original research on nurses’ PTG, and studies conducted after January 2020. Of 1089 identified articles, 142 were screened, and 27 were selected for final analysis. Data extracted from the articles included the author(s) name(s), the study’s geographic location, publication year, study purpose, study design, participants, methods, measurement scales, results, and notes. PTG factors were extracted and grouped into 4 broad categories: COVID-related factors, nursing-related factors, factors in Tedeschi’s PTG conceptual model, and other factors. Smaller categories were then created by inductively categorizing the factors based on similarities and differences.

**Results:**

As factors of nurses’ PTG during the early stage of the COVID-19 pandemic, 16 subcategories were organized under 4 categories. In addition to all factors from Tedeschi’s PTG model, some COVID-related factors (eg, care context, organizational training), and some nursing-related factors (eg, work environment) were shown to be related to PTG. No significant relationships were found between almost all of the other factors, including sociodemographic attributes, and PTG.

**Conclusions:**

Factors found in this study can help identify nurses’ PTG facilitators and guide the development of interventions for future crises.

## Teaser

Key points

- **What is already known on this topic:** During the COVID-19 pandemic, nurses working on the frontline were under psychological and physical stress, and these psychological and physical stresses not only reduced individual well-being and job satisfaction and increased turnover intentions, but also decreased workplace safety and patient quality of care. In contrast, positive psychological change—posttraumatic growth (PTG)—during pandemic situations could enhance occupational health such as work engagement of health care workers. Identifying factors that promote nurses’ PTG during infectious disease outbreaks may help nurses to recognize positive outcomes from traumatic experiences and disasters. However, no study has comprehensively identified factors affecting nurses’ PTG during a COVID-19 pandemic.
- **What this study adds:** This study found factors related to nurses’ PTG: all factors from Tedeschi’s PTG model (eg, personal resources, resilience, rumination), some COVID-related factors (eg, care context, organizational training), and some nursing-related factors (eg, work environment). No significant relationships were found between almost all of the other factors, including sociodemographic attributes (eg, age, sex, having children).
- **How this study affects research, practice, or policy:** Using these findings as a guide to improve the clinical practice environment for nurses can increase nurses’ PTG and contribute to building disaster-resilient health care systems. In future, the results of this study could potentially be applied as an evaluation criterion to connect nurses’ trauma to PTG during other disasters.

## 1. Introduction

In late 2019, the novel coronavirus, severe acute respiratory syndrome coronavirus 2 (SARS-CoV-2), spread from Wuhan, China, and on March 11, 2020, the World Health Organization^1^ declared it a pandemic. The global pandemic caused by the SARS-CoV-2 virus was a distressing and potentially traumatic experience for many,^2^^,^^3^ with frontline health care providers especially experiencing a significant impact. Health care providers faced numerous psychological challenges during the pandemic, including anxiety, depression, stress, insomnia, posttraumatic stress disorder (PTSD), psychological distress, burnout, and physical impact.^4^

Frontline nurses in particular were affected psychologically by the pandemic, given their greater visibility and accessibility within the health care team.^5^^-^^11^ A meta-analysis found that nurses who provided direct patient care experienced high levels of stress and had a higher prevalence of PTSD than other health professionals during the COVID 19 pandemic.^12^ Early in the pandemic, nurses struggled to provide standard care^13^ while facing a higher infection risk than other health care providers.^14^ Foli et al^15^ reported the traumatic experiences of frontline nurses as being like a “tsunami of death,” being “ torn between two matters,” and “betrayal.” A study of nurses at a tertiary care hospital who provided direct patient care during the COVID-19 pandemic found that about half of the nurses had clinical depression.^16^ Therefore, during the COVID-19 pandemic, nurses may have experienced more traumatic events and suffered both physically and mentally to a greater extent compared with other health care professionals. The prevalence of stress-related mental disorders among nurses has been linked to a decline in their personal well-being, diminished job satisfaction, and increased turnover intentions.^17^^,^^18^ These disorders have been identified as significant contributors to reduced workplace safety and compromised quality of patient care.^18^^,^^19^ Despite these negative effects, some nurses experienced personal and professional growth through caring for patients with COVID-19.^20^

The positive consequences of traumatic experiences have attracted increasing interest in recent years. One example is posttraumatic growth (PTG).^21^ PTG is defined as “positive psychological changes experienced as a result of the struggle with trauma or highly challenging situations.”^22^ PTG is characterized by positive changes in self-perception, interpersonal relationships, and philosophy of life, leading to increased self-awareness and self-confidence, a more open attitude toward others, a greater appreciation of life, and the discovery of new possibilities.^21^ Systematic reviews have revealed that 53% of individuals who have experienced trauma undergo PTG,^23^ exhibiting elevated levels of life satisfaction, happiness, and psychological, emotional, and even physical well-being.^24^ Additionally, research with health care providers has shown that PTG after a disaster increased subsequent job engagement.^25^ Given that nurses experience higher levels of stress and PTSD than other health care professionals, identifying factors that promote PTG after experiencing a traumatic event during a pandemic could help nurses to recognize not only negative psychological consequences but also positive consequences from traumatic experiences in future pandemics and disasters.

As the importance of PTG increases, research findings focused on PTG factors for nurses and similar human services are accumulating. Key factors contributing to PTG in nurses who have experienced patient loss or secondary trauma include age, female sex, positive self-compassion, compassion satisfaction, wisdom, deliberate rumination, motivation to work in the department, job satisfaction, work environment, and sufficient time to accept the event.^26^^-^^30^ Furthermore, several studies have been conducted of factors affecting PTG in human services. Studies of firefighters have identified a range of factors that contribute to PTG, including internal factors (self-elasticity, resilient elasticity, self-esteem), external factors (sense of calling, career calling), and coping mechanisms (problem-focused coping, intentional rumination).^31^^,^^32^ A study of general volunteers in the wake of the Great Earthquake in Japan identified team-building activities, prior psychological education, team collaboration, tailored family support, and recreation as key factors in promoting resilience.^33^ Various factors related to PTG in nurses have been presented; however, few studies have examined factors related to PTG among nurses during the COVID-19 pandemic. Nurses working on the frontlines of COVID-19 had higher PTG scores than nurses working in ICUs during a non-pandemic.^34^ This indicates that the degree of the effect on PTG varies depending on the type of trauma. Previous literature has categorized factors affecting PTG in nurses as individual, workplace, and social levels.^34^ In the context of concepts for which theoretical models have already been developed, it may be advantageous to categorize and differentiate between fundamental factors, as incorporated within the model, and those that are contingent upon situational factors for clarifying characteristics of the target population and the traumatic events.

The COVID-19 pandemic created unique challenges due to the rapid spread of the virus, evolving guidelines, shortages of medical resources and personnel, and emotional exhaustion for many nurses. Although 1 review study addressed the factors contributing to the PTG of health care providers, including nurses, during the COVID-19 pandemic,^35^ only 6 studies targeting nurses were included. As health care services are provided through multidisciplinary collaboration, the study of PTG among health care providers as a whole is essential. However, organizing the results to take into account the occupational characteristics of nurses is important. For example, nurses’ PTG scores generally tend to be higher than those of other health care providers, and a nurses-specific analysis is warranted.^36^^-^^38^ In addition, nurses had more frontline client contact in the delivery of care during the COVID-19 pandemic, and they reported being more aware of stress than other health care providers,^39^^,^^40^ which may have influenced their perception of the event as a traumatic event.

Therefore, we believe that a more comprehensive review targeting nurses is required. This literature review aimed to summarize factors affecting nurses’ PTG during the early stages of the COVID-19 pandemic. Given the long-term effects of COVID-19 and the possibility of significant differences in the social context and health care resource status at different phases, the current review included studies published up to February 2023 to capture the characteristics of the pandemic’s early stages. Clarifying PTG factors for nurses will stimulate the identification of PTG factors for other health care professions and allow for comparisons between professions. Comparisons of PTG factors between nurses and other health care professions may reveal PTG factors specific to each profession and PTG factors common to all professions. Understanding these factors will allow for effective support strategies for health care professionals.

## 2. Methods

This literature review followed the Preferred Reporting Items for Systematic Reviews and Meta-Analyses extension for Scoping Reviews (PRISMA-ScR).

### 2.1. Searching for relevant studies

A literature search was conducted in February 2023 using Medline, Cumulative Index to Nursing and Allied Health Literature (CINAHL), APA PsycINFO, Web of Science, and Google Scholar databases, using combinations of the following controlled terms: “Posttraumatic growth” or “Post-traumatic growth” and “COVID”. Articles published in English between January 2020 and February 2023 that reported on nurses’ PTG during the COVID-19 pandemic were considered for inclusion in the study.

### 2.2. Selecting appropriate studies

Inclusion criteria for the articles were as follows: (1) original and written in English, (2) focused on nurses’ PTG, and (3) reported studies conducted after January 2020. Exclusion criteria were as follows: (1) focused on participants other than nurses, (2) focused on areas other than PTG, (3) literature reviews, and (4) other reasons such as full text not being available. Studies involving both nurses and non-nurse attributes were included. To avoid missing articles, at the title and abstract screening stage we first extracted all articles in which health care professionals were participants and then excluded articles that did not include nurses as participants. Screening of the literature was conducted by 4 members of the research team in accordance with the criteria. If a decision could not be made, it was discussed and decided within the 4-person research team.

**Figure 1:**
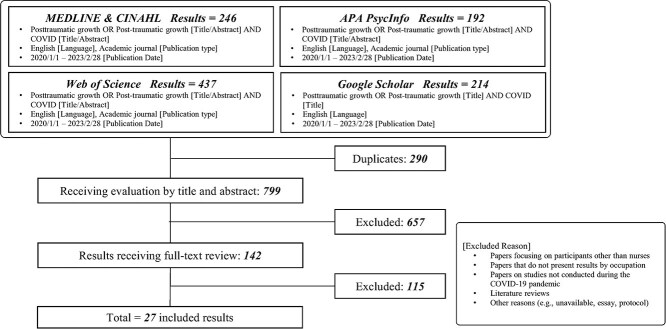
Search flowchart.

The initial literature search yielded 246 articles in Medline and CINAHL, 192 in APA PsycINFO, 437 in Web of Science, and 214 in Google Scholar. After excluding duplicates, the titles and abstracts of 799 articles were screened and matched with the inclusion criteria, yielding 142 articles. After reviewing the full texts of the 142 articles, 27 met the inclusion criteria. In total, 27 articles^15^^,^^41^^-^^66^ were identified as relevant to the review. Details of the screening process are shown in Figure 1.

### 2.3. Data extraction and synthesis

Data from the relevant articles were extracted in extraction format under the following subheadings: author(s) name(s), study’s geographic location, publication year, study purpose, study design, study participants, methods, measuring scales, results, and notes. For studies that included both nurses and non-nurses, we extracted nurse-only data when possible, or total data if nurse-only data were not separated. For 1 study where nurse-only data were not separated, we considered the data to be representative of nurses owing to the large proportion of nurses included in this study (80.0%).

Posttraumatic growth factors were then extracted and grouped into categories with reference to Tedeschi’s PTG conceptual model.^22^ Factors not included in the conceptual model were classified inductively based on similarities and differences, and categories were created and named. For the qualitative studies, the themes and categories related to PTG extracted were organized separately from the quantitative studies. These data extractions were performed blindly by 2 researchers (K.S. and K.I.) and verified by 1 researcher (S.N.) when results did not agree. When necessary, decisions were discussed within the 4-person research team. Categorization was mainly performed by K.S., and verified by the remaining 3 team members (K.I., S.N., Y.O.). The whole research team repeatedly discussed and refined the classification and category titles.

## 3. Results

### 3.1. Characteristics of the reviewed articles

Of the 27 articles, 22 were quantitative, 4 used a qualitative approach,^15^^,^^44^^,^^53^^,^^54^ and 1 used a mixed-methods approach.^62^ Twenty-five studies were observational and 2 were experimental. All observational studies were cross-sectional. Eighteen studies (66.7%) investigated the prevalence and determinants of PTG in nurses, and 3 (11.1 %) examined nurses’ experiences during the COVID-19 pandemic in general, including PTG, and 2 (7.4%) evaluated the effects of interventions on PTG. In all the literature where settings were mentioned, the setting was a medical institution. Many studies were conducted through online survey and some had unclear settings (Appendix).

Table 1 shows the distribution of the studies by publication year and study location. Most studies were conducted in China (35.7%), followed by Turkey (25.0%). The overall number of studies of PTG among nurses is increasing, and an increasing number of studies in countries other than China have contributed to this trend.

**Table 1:** Study location and publication year (numbers refer to the References list).

**Location**	**2020**	**2021**	**2022**	**2023**	**Total** ^**a**^
**United States**		14	42, 54	51	4
**Australia**			33		1
**Israel**			40		1
**Kosovo**		52			1
**Turkey**			34, 36, 37, 43, 53, 57	35	7
**China**	38, 39	47, 48, 50, 58	44, 45, 49, 55		10
**South Korea**	46		41, 56		3
**Taiwan**	38				1
**Total** ^**a**^	4	6	16	2	28

a One study was conducted in 2 countries.

### 3.2. Definition and scale of PTG in the reviewed articles

Of the included studies, 15 used Tedeschi’s definition of PTG,^41^^,^^42^^,^^44^^,^^46^^-^^49^^,^^51^^,^^53^^,^^56^^,^^57^^,^^60^^,^^62^^-^^64^ and 3 used the definition from the literature citing Tedeschi.^52^^,^^55^^,^^66^ Six studies did not provide a definition with citation^43^^,^^50^^,^^58^^,^^59^^,^^61^^,^^65^; however, they were assumed to follow Tedeschi’s definition based on the scale they used. The remaining 3 were qualitative studies and did not include a definition.^15^^,^^45^^,^^54^

Five studies mentioned using the PTG Inventory (PTGI)^21^ as a measure of PTG; 13 used a translated language version of the PTGI—5, 5, 2, and 1 used the Turkish, Chinese, Korean, and Hebrew versions, respectively. Of the 5 studies that reported using the original PTGI, 2 may have used the Chinese version of the PTGI, as it is used for other measurement scales. The Chinese and Korean versions of the PTGI differ from the original regarding the number of scale items, whereas the Turkish version has a different subscale structure.

Two studies used the PTG Inventory-Short Form (PTGI-SF),^67^ a shortened version of the PTGI, and 1 study used the PTG-X (PTGI-X),^68^ an expanded version of the PTGI. One study did not specify which scale was used.

### 3.3. Factors of nurses’ PTG

Factors affecting PTG during the COVID-19 pandemic were classified into “COVID-related factors,” “nursing-related factors,” and “other factors,” in addition to “PTG model factors,” creating a total of 4 categories. Table 2 shows the factors affecting nurses’ PTG. Patient care, care context, organizational training, health issues, and loss were included as subcategories of COVID-related factors. Professional background, employment, and work environment were included as subcategories of nursing-related factors. Personal resources, traumatic events, psychological pain, deliberate rumination, self-disclosure, control, resilience, and sociocultural impact were included as subcategories of known concepts within the PTG model. Other factors included were age, sex, educational status, marital status, having children, income status, country of birth, mental health, subjective well-being, satisfaction with life, and transformative power of pain.

**Table 2:** Factors affecting posttraumatic growth (numbers refer to the References list).

**Factors of posttraumatic growth**	**Significant positive association**	**Significant negative association**	**Nonsignificant**
**COVID-RELATED FACTORS**			
***Patient care***			
Worked in a COVID-19-designated hospital			46
Worked in a COVID-19-related department	55		42, 46
Caring for a patient with COVID-19	46		41, 42, 51, 60,
Caring for a patient with COVID-19 who died			42, 46
Period of caring for patients with COVID-19	60	44	42
Increase in working hours during pandemic			51
***Care context***			
Working in Hubei	52		
Working in Istanbul	44		
Stayed at hotels (vs hospital public housing, home/family)	44		
Insufficient PPE resources (vs enough, unsure)		44	
No communication problems with the health care team (vs sometimes, usually)	44		
***Organizational training***			
Compassion and growth workshop	59		
Psychological intervention or training	47		
Psychoeducational group programme	62		
***Health issues***			
Pandemic-related stress	41		
Having had COVID-19 infection			42, 51
Physical discomfort during the pandemic	58		
Feeling the need for psychiatric or psychological help after coronavirus	61		
Having a family member with COVID-19 infection			42, 61
Worry about getting infected with COVID-19	63		
Worry about family members getting infected with COVID-19	63		
***Loss***			
Losing a family/relative member due to COVID-19	42		51
Losing a health care worker relative due to COVID-19	51		
Experiencing economic loss due to COVID-19			42
Experiencing social loss due to COVID-19			42
**NURSING-RELATED FACTORS**			
***Professional background***			
Work experience (years of experience/tenure)	46, 47, 48		41, 42, 51, 60, 61, 63
Working year as an infection control nurse	51		
Professional title		66	
Administrative			48
Professional self-identity	57		
***Employment***			
Working mode—full time		63	
Night work (vs daytime/shift work)	42		61
Working hours	44		
Number of visits to patient room per day	44		
Hospital/organization type			61
Working unit			61
Worked in critical care units			46
Having direct patient care responsibilities	59		
***Work environment***			
Awareness of risk	47		
Self-confidence in frontline work	47		
Came from other province to support	57		
Satisfaction with workplace pandemic control guidelines			63
Work satisfaction	63		
**FACTORS IN THE PTG MODEL**			
***Personal resource***			
Self-compassion	41		
General self-efficacy	66		
Having a religious affiliation	63, 49		
Religiosity	48		

**Table 2:** Continued.

**Factors of posttraumatic growth**	**Significant positive association**	**Significant negative association**	**Nonsignificant**
***Traumatic event***			
Trauma	46		
Trauma events	49		
***Psychological pain***			
Burnout	46		
Posttraumatic stress disorder	43, 52, 64	66	
Posttraumatic stress			50
Depression		41	
Anxiety			41
Psychological distress	63		
***Deliberate rumination***			
Deliberate rumination	47, 49, 64		
***Self-disclosure***			
Self-disclosure	49		
***Control***			
Coping skill	60		
Coping style	66		
Meaning in life	49		
***Resilience***			
Resilience	44, 49, 56		43
***Sociocultural impact***			
Social support	49, 57, 58, 64, 66		
***Other factors***			
Age		61	42, 48, 51, 55, 63
Gender—female (vs male)		61	46, 48, 51, 55
Educational status			42, 51, 55, 61
Marital status—married (vs unmarried)	49		42, 51, 61, 63
Having children	58		42, 51, 55, 61, 63
Income status			61
Birth country—other country (vs Israel)			48
Mental health			60
Subjective well-being	41		
Satisfaction with life	42		
Transformative power of pain	61		

Abbreviation: PPE, personal protective equipment.

Regarding COVID-related factors, numerous studies have examined factors related to patient care, with some reporting that patient care was a factor that increases PTG. In the context of care, working in an infection-endemic area, communication with the health care team, and adequate personal protective equipment (PPE) resources were reported as factors that increased PTG.

Psychoeducational programmes provided by researchers and organizations have also effectively promoted PTG. In terms of health problems, psychological distress, such as stress, fear of infection, and physical discomfort, were associated with higher PTG. Loss of family and other health care providers was associated with higher PTG.

As for nursing-related factors, many studies have examined their association with work experience, with one-third reporting higher PTG with more work experience. Regarding working conditions, several studies have reported a positive association between working hours and night shifts and PTG levels. Regarding the working environment, human resource support and job satisfaction were positively associated with PTG.

Concepts within the PTG model, such as psychological pain, deliberate rumination, self-disclosure, control, resilience, and sociocultural support, have been tested as factors in many studies, most of which have shown positive associations and support the model.

Regarding other factors, many studies examined associations with participant demographics, such as age and gender; however, most studies did not show statistically significant differences. Psychological resources such as well-being and life satisfaction were positively associated with PTG.

### 3.4. Nurses’ experience extracted as PTG during COVID-19

Four studies qualitatively explored the experiences of nurses caring for patients with COVID-19. All studies had a growth component as one of their themes, such as resilience/PTG through self and others^15^ and taking another step in one’s growth.^54^ Subthemes of growth included care-related content, such as professional development^43^ and providing real nursing^54^.

Two of the 4 studies examined the experiences of nurses infected with COVID-19 and included specific elements such as stigma and appreciation of life^45^ as subthemes.

## 4. Discussion

This review identified 4 categories of factors influencing PTG in nurses during the COVID-19 pandemic: COVID-related factors, nursing-related factors, factors in the PTG model, and other factors. These categories highlight factors specific to nurses during the COVID-19 pandemic and may be useful when discussing PTG in other disasters and professional settings.

### 4.1. Classification of PTG factors

The 4 classifications we used in this review proved instrumental in identifying elements applicable to future research on unknown disasters, thus underscoring the practical relevance of these findings. This classification is unique in that it allows the identification of PTG factors that reflect the characteristics of the target population and the traumatic event by making the factors included in the most commonly used PTG models independent. Previous systematic reviews of PTG in nurses included a wide variety of traumatic events, such as disasters, violence in health care settings, and patient deaths, and categorized the factors associated with PTG in nurses as individual, work, and social/organizational-related factors.^34^ However, these classifications were abstract, making it difficult to find the intervention points. Additionally, different events could yield markedly different patterns of PTG domains and posttraumatic stress.^69^ Existing classifications may bury the characteristics of the target population and the traumatic events. The significance of this study’s classification lies in its potential to facilitate comparative research and its applicability to nursing practice by clarifying these characteristics. For example, categorizing PTG factors enables a clearer comparison of the characteristics and impacts of COVID-19 and other pandemics. This approach aids in identifying both the unique and shared factors across different disasters, thereby enhancing our understanding of their underlying mechanisms. Additionally, recognizing PTG factors specific to each disaster allows for the design of more effective interventions. For instance, the prolonged psychological burden observed during the COVID-19 pandemic, marked by stress and fear of infection, underscores the need for long-term stress management interventions. Conversely, the relatively short duration of severe acute respiratory syndrome (SARS) outbreak^70^ suggests that acute stress-coping strategies may be more appropriate. Therefore, interventions tailored to each pandemic’s unique features should be designed more effectively. Moreover, understanding the specific PTG factors that nurses encounter during the pandemic will enable the development of more precise support strategies for nurses, which may also be useful when discussing support strategies for other health care professionals. Both resilience and social support were crucial to fostering PTG during COVID-19 and SARS among nurses. By tailoring support strategies to each disaster’s specific demands, nurses’ mental health and well-being can be better supported. Overall, establishing a classification framework for PTG factors allows future studies to adapt to different pandemics or disaster patterns, thereby promoting a comprehensive understanding and response. This approach advances academic enquiry and practical applications in nursing and disaster management.

### 4.2. Definition of traumatic events

This review’s findings indicate that many studies lacked a clear definition of what constitutes a “traumatic” event, which can lead to inconsistencies in the interpretation and measurement of PTG. Therefore, future studies should establish a clear definition of trauma, which could result in more nuanced and consistent interpretations of growth.

This review classified COVID-related factors into subcategories such as patient care, organizational training, health issues, and loss, thus broadening our understanding of the diverse range of factors contributing to PTG, including those previously difficult to identify due to their unique traumatic characteristics. This refined categorization not only clarifies the variation in factors but also underscores the importance of using a consistent classification framework in future studies. Li et al^71^ suggested that employing a consistent classification framework for findings across studies, rather than allowing individual researchers to develop their own, would improve the integration of findings and enhance the comparability and synthesis of future research results.

### 4.3. Characteristics of factors of PTG

We found that few studies (eg, Yim and Kim^64^) were based on the PTG theory model of Tedeschi et al.^22^ In addition, some studies examined related factors in a direction opposite to the theoretical model. Because many studies use the scales developed by Tedeschi et al, future studies should reference the original theoretical model when investigating related factors. Future studies should aim to conduct studies based on this theory and explore new perspectives, potentially contributing to the development of the PTG theory.

Moreover, the participant characteristics presented in previous studies, such as age, female sex, and educational background, did not show significant associations with PTG among nurses during the early stages of the COVID-19 pandemic. However, prior studies have indicated that these attributes influence PTG. This study suggests that rather than these individual characteristics, factors related to events caused by trauma and those associated with the nursing profession may significantly foster PTG among nurses. The extracted COVID-related factors can be interpreted as situation-specific elements that embellish the concepts in Tedeschi’s theoretical model.^22^ For example, factors in the “loss” category could be an additional qualifier to the traumatic event. In addition, psychological distress included in health problems is similar to psychological pain or stress as part of the theoretical model and can be interpreted as part of the process of PTG. As mentioned by Tedeschi, PTG and stress are mutually influential and important comorbid factors.^22^ These factors can be applied to future events by reflecting the characteristics of the event. In addition, care-related factors indicate more demanding work, leading to the stimulation of social support and increased competence, which may be associated with higher PTG. This may also be the reason why working in infection-endemic areas such as Hubei was identified as a factor. In addition, some of the nurses who worked in the infection-endemic areas were dispatched from other areas, and it is said that nurses’ sense of mission and the pride of being selected for the dispatch process may have contributed to enhancing PTG.^52^ The extraction of an association with work factors is consistent with the findings of other reviews.^34^^,^^72^ However, even with the same factors, some studies showed a positive association, whereas others did not. Future studies should use more precise methods to promote PTG by identifying variables (confounders) that lead to differences in these associations.

The results of this study may be useful in studying PTG in other medical professionals. Previous studies have suggested reasons why nurses may have higher PTG than other medical professionals, such as their familiarity with help-seeking behavior, their ease in obtaining social support owing to their large population, and their increased opportunities for exposure to secondary traumatic stress in patient care.^36^^-^^40^^,^^73^^,^^74^ When conducting PTG research with other health care professionals, these traits should be applied with consideration of their possible association with resilience and social support.

### 4.4. Characteristics of experimental studies

Only 2 of the 27 studies reviewed were experimental studies, highlighting a significant gap in the availability of interventions and support for nurses during pandemic situations. PTG and resilience are similar concepts related to the positive aftermath following a crisis,^75^ and interventions can enhance nurses’ resilience.^76^ Furthermore, studies of oncology nurses have shown that interventions such as lectures, reading materials, video demonstrations, and exercises can enhance PTG.^77^ However, previous studies have not established support systems or methods to transform nurses’ traumatic experiences from pandemics and disasters to PTG. Future studies should develop interventions aimed at creating support methods that help nurses who often work on the frontlines during pandemics and disasters connect trauma experiences to psychological growth after experiencing traumatic events.

### 4.5. Need for long-term evaluation of PTG

Of the 22 quantitative studies and 1 quantitative part of the mixed-methods study reviewed, 21 were cross-sectional. PTG occurs after a traumatic event, and some reports suggest that it can take years for a person to perceive it. Furthermore, nurses’ PTG can change over time, particularly during the COVID-19 pandemic.^3^^,^^78^ However, all studies included in this review were conducted during the early stage of the COVID-19 pandemic. Future studies should clarify the starting point of the traumatic event and adopt a longitudinal design to measure changes in PTG. Additionally, planning research designs that consider the characteristics of PTG, such as asking about PTG after the outbreak has subsided, is ideal.

### 4.6. Limitations

This review has some limitations that should be considered when interpreting its findings. First, this review excluded gray literature, which may have limited the comprehensiveness of the findings. The exclusion of non–peer-reviewed studies could mean that relevant data were not considered, potentially influencing the overall interpretation of the factors contributing to PTG. Second, the studies reviewed were conducted in countries or regions where the pandemic was at different stages at the time of the research. The different stages of the pandemic, when trauma occurred, and when PTG was measured, make it difficult to interpret the results between studies. Therefore, factors influencing PTG were also possibly different owing to these different pandemic conditions. Future research should compare studies under more controlled conditions, such as matching the regional situation and the timing of the start of the study. Finally, this review acknowledges the difficulties associated with studying COVID-19 trauma, particularly regarding the timing of trauma and measurement of PTG. These challenges stem from the dynamic nature of the pandemic and the variability in how and when trauma is experienced and assessed across different studies. Despite these complexities, this review effectively identified and categorized the factors influencing PTG amid the diverse and evolving conditions of the pandemic.

## 5. Conclusions

This review organized factors contributing to PTG into 4 categories: COVID-related factors, nursing-related factors, factors in Tedeschi’s PTG conceptual model, and other factors, suggesting factors specific to nurses in COVID-19 and factors that may be common to other disasters and professions. These findings may help identify factors promoting PTG and develop intervention programmes leading to PTG in nurses for future disasters.

## 6. Relevance for clinical practice

The review identified several factors that increased PTG, including the context of care during an infectious disease epidemic, such as communication with the health care team and adequate PPE resources, psychological education programmes, and the work environment, such as staff support and job satisfaction. Countries worldwide faced PPE shortages during the global pandemic. Ensuring the safety of frontline health care workers during a crisis not only guarantees their safety and security but may also contribute to their sense of growth. Therefore, increasing protective equipment stocks and establishing supply systems is necessary. Using these findings as a guide to improve the clinical practice environment for nurses can increase nurses’ PTG and contribute to building disaster-resilient health care systems.

Additionally, recognizing the complex relationship between trauma severity and growth is essential. These findings should not be interpreted to suggest that greater trauma leads to greater growth. Instead, emphasis should be placed on preventing trauma whenever possible. This review did not focus on studies examining preventable trauma; however, it provides a framework for understanding the factors that contribute to PTG when trauma is unavoidable.

In the future, the results of this study could potentially be applied as an evaluation criterion to connect nurses’ trauma to PTG during other disasters. However, since many of the studies were observational and focused on individual attributes, future research should identify social and environmental factors as well as the characteristics of hospitals and wards that contribute to PTG. Additionally, the results were obtained during the pandemic; therefore, caution is needed when applying these findings. Future research should use longitudinal designs to identify factors that influence PTG several years after an infectious disease outbreak has subsided. Moreover, examining how PTG evolves could provide valuable insights for supporting individuals who have experienced traumatic events.

## Supplementary Material

Web_Material_uiaf030

## Data Availability

Data sharing is not applicable to this article as no new data were created or analysed in this study.
