# Supplementary material for: Factors related to nurses’ posttraumatic growth during the early stage of the coronavirus disease 2019 pandemic: a scoping review
Source: J Occup Health. 2025 May 26;67(1):uiaf030. doi: 10.1093/joccuh/uiaf030 (PMC12229266; doi:10.1093/joccuh/uiaf030)
Supplement: Web_Material_uiaf030 [file web_material_uiaf030.zip › COVID review_appendix.pdf]

Appendix. List of references included in the review

| First author (Year), Location | Purpose studied | Design & Participants | Data collection | Definition of PTG | Scale for measuring PTG | Main Analysis |
|---|---|---|---|---|---|---|
| Aggar, C. (2022)[41], Australia | To explore the impact of pandemic-related stress on psychological adjustment outcomes and potential protective factors for nurses working in the Australian acute care sector during the COVID-19 pandemic. | Cross-sectional, Quantitative, 767 nurses | Sep-Nov 2020 | The perception of positive outcomes and personal benefits following highly stressful events (Tedeschi et al, 1996) | Posttraumatic Growth Inventory–Short Form (Cann et al. 2010) | Hierarchical multiple regression analyses |
| Aker, M. N. (2022)[42], Turkey | To examine the relationship between PTG and satisfaction with life | Cross-sectional, Quantitative, 517 nurses | Apr-Jun 2021 | Positive psychological changes that may occur after the individual struggles with difficult or negative events (Tedeshi et al, 2018) | | T-test, one-way ANOVA, correlation |
| Ata, E. E. (2023)[43], Turkey | To determine the relationship among resilience, PTSD, and PTG | Cross-sectional, Quantitative, 192 nurses | Sep-Dec 2021 | | Turkish version of PTGI (Kagan et al, 2012) | Whitney-U test, Kruskal-Wallis test, correlation |
| Atay, N. (2022)[44], Turkey | To identify the factors of PTG | Cross-sectional, Quantitative, 263 nurses | Jun-Sep 2020 | The ability of an individual to move beyond her adaptation functionality, awareness,beliefs, and goals that have been shattered by a traumatic event (Tedeshi et al, 2018) | Turkish version of PTGI (Kagan et al, 2012) | Whitney-U test, Kruskal-Wallis test, correlation |
| Aydin, R. (2021)[45], China | To explore the experiences of nurses diagnosed with COVID-19 | Qualitative, 18 nurses diagnosed with COVID-19 | Jan 2021 | | N/A | Thematic analysis |
| Chen, R. (2020)[46], China, Taiwan | To assess trauma, burnout, posttraumatic growth, and associated factors for nurses in the COVID-19 pandemic | Cross-sectional, Quantitative, 12,596 nurses | Apr 2020 | Positive psychological change experienced as a result of the struggle with highly challenging life circumstances (Tedeschi et al, 2004) | Posttraumatic Growth Inventory–Short Form (Cann et al. 2010) | Multiple linear regression<br>Multiple logistic regression |
| Cui, P. P. (2020)[47], China | To explore the level and influencing factors of frontline nurses' post- traumatic growth (PTG) during COVID-19 epidemic | Cross-sectional, Quantitative, 167 nurses | Feb 2020 | Positive psychological changes after an individual experiences traumatic events (Tedeshi et al, 1990) | Simplified Chinese version of PTGI (Wang et al, 2011) | Multiple linear regression |
| Dahan, S. (2022)[48], Israel | To measure the level of anxiety, concern, resilience, and PTG | Cross-sectional, Quantitative, 183 nurses | Apr 2020 | Positive change that occurs as a result of the struggle with highly challenging life crisis (Tedeshi et al, 2004) | Hebrew version of PTGI<br>(Laufer et al, 2006) | Hierarchical regression analysis |
| Foli, K. J. (2021)[15], US | To describe the experiences of frontline nurses who are working in critical care areas during the COVID-19 pandemic with a focus on trauma and the use of substances as a coping mechanism | Qualitative, 105 nurses | Jun-Sep 2020 | | N/A | Content analysis |
| Han, S. (2022)[49], South Korea | To identify the factors of PTG | Cross-sectional, Quantitative, 233 nurses | May-Jul 2021 | Positive psychological change due to coping with or fighting stressful events (Tedeschi et al, 1996) | Korean version of PTGIX<br>(Kim et al, 2020) | Hierarchical regression analysis |
| Hickling, M. T. (2022)[50], US | To investigate mental health reactions to dealing with COVID-19 | Cross-sectional, Quantitative, 112 nurses | summer-fall in 2020 (2 months) | | Post-traumatic Growth Inventory (Tedeschi et al, 1996) | Multiple hierarchical regression |
| İnce, S. Ç. (2022)[51], Turkey | To measure the level of PTG | Cross-sectional, Quantitative, 170 nurses | Feb 2020 | A significant positive mental change in the life of a person who survived a forcing or traumatic event (Tedeschi et al, 1996) | Turkish version of PTGI<br>(Kagan et al, 2012) | Whitney-U test, Kruskal-Wallis test |
| Jiang, H. (2022)[52], China | To compare the difference of PTSD, professional benefits, and PTG between working inside and outside Hubei | Cross-sectional, Quantitative, 3,419 nurses | Feb 2020 | Positive psychological changes that individuals experience after experiencing life crisis (Silverstein et al, 2018) | Simplified Chinese version of PTGI (Wang et al, 2011) | T-test<br>Multiple logistic regression |
| Jiang, J. (2022)[53], China | To explore the experience and the process of PTG | Qualitative, 13 nurses diagnosed with COVID-19 | Jun 2022 | Positive psychological changes that occur after an individual experiences traumatic events (Tedeshi et al, 1996) | N/A | Phenomenological |
| Lee, N. (2020)[54], South Korea | To explore the meaning and nature of the patient care experiences | Qualitative, 18 nurses | Jun-Sep 2020 | | N/A | Phenomenological |
| Li, L. (2021)[55], China | To assess post-traumatic growth (PTG) of Chinese nurses and GP during the COVID-19 pandemic and protective factors contributingto PTG. | Cross-sectional, Quantitative, 4,555 nurses and 424 GPs | Mar 2020 | A significant positive change in an individual life as a consequence of exposure to a challenging or traumatic event (Bernstein et al, 2018) | Chinese version of PTGI<br>(Dong et al, 2013) | T-test<br>Multiple linear regression |
| Liu, X. (2021)[56], China | To examine the mediating effect of PTG and professional benefits between resilience and intent to stay | Cross-sectional, Quantitative, 266 nurses | Apr-May 2020 | Transformation following trauma (Tedeschi et al, 1996) | Simplified Chinese version of PTGI (Wang et al, 2011) | Structural Equation Modeling |
| Mo, Y. (2022)[57], China | To examine the relationship between PTG and professional self-identity and social support | Cross-sectional, Quantitative, 267 nurses | Feb-Apr 2020 | A significant positive change in an individual life as a consequence of exposure to a challenging or traumatic event (Tedeschi et al, 1996) | Post-traumatic Growth Inventory (Tedeschi et al, 1996)* | Multiple linear regression |
| Peng, X. (2021)[58], China | To identify the factors of PTG | Cross-sectional, Quantitative, 116 nurses | Apr 2020 | Resulting from coping with trauma and adaptive response to the adverse trauma | Simplified Chinese version of PTGI (Wang et al, 2011) | Multiple linear regression |

| | | | | | | |
|---|---|---|---|---|---|---|
| Pfeiffer, K. (2021)[59], US | To describe the effects of an intervention called "Compassion & Growth Workshops" on reported posttraumatic growth | Intervention (single, pre-post), Quantitative, 163 nurses and advanced practice nurses | Jan-Mar 2021 | | Posttraumatic Growth Inventory Expanded (Tedeschi et al, 2017) | Multiple logistic regression |
| Prekazi, L. (2021)[60], Kosovo | To explore the impact of coping skills in developing PTG among healthcare providers | Cross-sectional, Quantitative, 691 healthcare providers (including 550 nurses) | Jan 2021 | Positive life changes including an increased appreciation for life, meaningful interpersonal relationships, sense of personal strength, changed priorities, and a richer existential/spiritual life (Tedeschi et al, 1995 etc.) | Post-traumatic Growth Inventory (Tedeschi et al, 1996) | Hierarchical regression analysis, mediation analysis |
| Sarıalioğlu, A. (2022)[61], Turkey | To examine the relationship between the transformative power of pain and posttraumatic growth in nurses who are inevitably exposed to the pain of patients by providing health services. | Cross-sectional, Quantitative, 175 nurses diagnosed with COVID-19 | Apr-Aug 2021 | Positive experience of change and an increase in functionality after a major crisis in life | Turkish version of PTGI (Dürü, 2006) | Multiple linear regression |
| Sawyer, A. T. (2022)[62], US | To measure the feasibility and acceptability of the psychoeducational program<br>To determine its impact on PTG, resilience, insight, self-compassion, empowerment, perceived stress, burnout, and job satisfaction | Intervention (single, pre-post), Mixed method<br>19 nurse managers | May 2021 (baseline)<br>Jul 2021 (follow-up) | Positive psychological change experienced through the struggle with adversity, traumatic events or crises, such as the pandemic (Tedeschi et al, 2004). | Post-traumatic Growth Inventory (Tedeschi et al, 1996) | Paired T-test<br>Thematic analysis |
| Yeung, N. C. (2022)[63], China | To examine how socio-demographic characteristics, COVID-19-related worries, and work-related variables (satisfaction with work and workplace pandemic guidelines) were associated with PTG among nurses in Hong Kong<br>To examine moderate effect of current distress on this relationship. | Cross-sectional, Quantitative, 1,510 nurses | Aug-Sep 2020 | Positive changes that occur as a result of the struggle with highly stressful life events (Tedeshi et al, 2004) | | Hierarchical regression |
| Yim, J. Y. (2022)[64], Korea | To establish a path model of posttraumatic growth among nurses who provided care for coronavirus disease 2019 (COVID-19) patients and to examine the associations between the relevant variables. | Cross-sectional, Quantitative, 229 nurses | Apr-May 2021 | Positive changes that individuals experience (Tedeshi et al, 2004) | Korean version of PTGI (Song et al,2009) | Structural Equation Modeling |
| Yüceler Kaçmaz, H. (2022)[65], Turkey | To identify the factors of PTG | Cross-sectional, Quantitative, 559 nurses | Jan-Mar 2021 | After the traumatic experience, there may be positive changes in some areas | Turkish version of PTGI (Kagan et al,2012) | T-test, one-way ANOVA, correlation |
| Zhang, X. T. (2021)[66], China | To identify the factors of PTG | Cross-sectional, Quantitative, 1,790 nurses | Jun 2020 | Positive psychological change experienced as a result of the struggle with highly challenging life circumstances (Di Terra et al, 2021) | Post-traumatic Growth Inventory (Tedeschi et al, 1996)* | Multiple linear regression |

*: There is a high probability that the Chinese version was utilised; however, no information has been provided regarding the translated version.
